# Retrospective Analysis of Pneumonic Tularemia in Operation Whitecoat Human Subjects: Disease Progression and Tetracycline Efficacy

**DOI:** 10.3389/fmed.2019.00229

**Published:** 2019-10-22

**Authors:** Mark S. Williams, Marianne R. Baker, Tina Guina, Judith A. Hewitt, Lynda Lanning, Heather Hill, Jeanine M. May, Beverly Fogtman, Phillip R. Pittman

**Affiliations:** ^1^Office of Biodefense Research Resources and Translational Research, Division of Microbiology and Infectious Diseases, National Institute of Allergy and Infectious Diseases, National Institutes of Health, Bethesda, MD, United States; ^2^Office of Regulatory Affairs, Division of Microbiology and Infectious Diseases, National Institute of Allergy and Infectious Diseases, National Institutes of Health, Bethesda, MD, United States; ^3^The Emmes Company, Rockville, MD, United States; ^4^Department of Clinical Research, United States Army Medical Research Institute of Infectious Diseases, Fort Detrick, MD, United States

**Keywords:** tularemia, human, pneumonic, Whitecoat, tetracycline

## Abstract

*Francisella tularensis* is a highly infectious Gram-negative bacterium that is the etiologic agent of tularemia in animals and humans. The incidence of tularemia is very low with a lack of comprehensive data that describe disease in humans due to difficulty in understanding time and routes of exposure. Under the title Operation Whitecoat, researchers at Ft. Detrick, MD conducted 40 clinical studies of tularemia from 1958 to 1968. In these studies, one of the objectives was to evaluate candidate countermeasures for treatment or prophylaxis of disease after exposure to *Francisella tularensis* strain Schu S4 by inhalation. These studies were reviewed retrospectively to delineate the early signs and symptoms or natural history of pneumonic tularemia and examine the efficacy of tetracycline in controlled human clinical studies. Using vital signs, onset of fever was objectively defined and calculated for each subject, while Adverse Events reported after exposure were also used to define the timing of disease onset and symptoms of early disease. There was a dose response relationship between time to fever onset and exposed dose at 200 cfu (172.8 h), 700 cfu (163.2 h), 2,500 cfu (105.3 h), and 25,000 cfu (75.5 h). Onset of fever was typically the earliest sign of disease at all doses but was often accompanied by symptoms such as headache, myalgia, chest pain, and nausea, irrespective of dose except at 200 cfu where only 50% of subjects exhibited fever onset or symptoms. Examining the efficacy of different treatment regimens of tetracycline, ineffective treatments were indicated by relapse of disease (fever and Adverse Events) after cessation of antibiotic treatment. Stratification of the data suggested that treatment for <14 days or doses <2g/day was associated with increased percentage of subjects with relapse of disease symptoms. Although these types of human challenge studies would not be ethically possible now, the climate post-World War II supported human testing under rigorous conditions with informed consent. Thus, going back and analyzing these unique clinical human challenge studies has helped describe the course of infection and disease induced by a biothreat pathogen and possible countermeasures for treatment under controlled conditions.

## Introduction

*Francisella tularensis* is a highly infectious Gram-negative bacterium that is the etiologic agent of tularemia in animals and humans (1). Tularemia has been called rabbit fever, deer fly fever, and market men's fever in the United States; wild hare disease (yato-byo) and Ohara's disease in Japan; and water-rat trappers' disease in Russia. *F. tularensis* strains have been weaponized for potential use as a biothreat agent by several countries (2). Infections with highly virulent *F. tularensis* strains are lethal in up to 60% of individuals infected by the inhalation route if not treated with antibiotics (3). For these reasons, *F. tularensis* has been designated a Category A select agent by the Centers for Disease Control and Prevention (CDC) and the National Institutes of Health (NIH) (4).

Human cases of tularemia have been classified in six classic forms: ulceroglandular when skin ulceration and inflamed lymph nodes are present, glandular when inflamed lymph nodes exist without obvious skin ulceration, oculoglandular when eye involvement is present, pharyngeal when stomatitis and exudative pharyngitis or tonsillitis, abdominal pain, nausea, cervical lymphadenopathy, diarrhea, and gastrointestinal bleeding are present, typhoidal when no other route is obvious, and pneumonic, which includes the outcomes of infection by inhalation (5). Pneumonic disease is considered the most severe form of tularemia in humans and inhalation is the likely route of infection in an intentional release of *F. tularensis*.

The incidence of pneumonic tularemia in the United States and worldwide is very low, therefore, it is not feasible to conduct clinical efficacy testing of tularemia medical countermeasures (MCM) in humans. During 2001–2010, a total of 1,208 cases of all forms of tularemia were reported; the median number of cases per year was 126.5 with a range of 90–154 cases per year. These cases typically were spread out geographically and remained sporadic in nature, although small outbreaks have been reported (6). Of these 1,208 reported cases, 64% were categorized as confirmed and 35% as probable. Average annual incidence was 0.041 cases per 100,000 persons (6). Even in the situations where clinical data have been reported (6–9), describing disease course is difficult due to lack of understanding of timing and route of exposure. Thus, there is an inability to conduct human efficacy trials for tularemia treatments and there is a lack of comprehensive data that describe the course of pneumonic tularemia in humans.

Therefore NIAID, in partnership with the government agency BARDA, are working with the FDA to establish an animal model for pneumonic tularemia to be used for medical countermeasure testing under the Animal Rule (10). Specifically, the efforts are to qualify the cynomolgus macaque model for pneumonic tularemia (11) in the FDA's Animal Model Qualification (AMQ) program, which is one of the Drug Development Tools Qualification Programs (12). An integral part of the Animal Rule and the AMQ process is to ensure that the animal species reacts and responds to infection in a manner that is predictive for humans. Given the difficulty in obtaining data on tularemia in humans, it was decided to gain access to and analyze controlled human studies of pneumonic tularemia that were performed post-World War II during the Operation Whitecoat Clinical Studies as described below.

Human clinical testing of countermeasures for pneumonic tularemia were initiated by the Department of Defense (DoD) in the 1950's through the Commission of Epidemiologic Survey (CES) under the Armed Forces Epidemiology Branch (AFEB) (13). Importantly, these studies were performed with strict policies for patient safety and a three-step process of informed consent and voluntary participation. DoD-supported studies were performed at Ohio State University (14) and University of Maryland School of Medicine (15). Saslaw et al. (14), reported initial human exposure studies with human subjects exposed to low levels of Schu S4 (10–50 cfu) and described that the initial presentation of pneumonic tularemia could be like a systemic illness in the absence of signs of respiratory disease. Similarly, McCrumb (15) exposed subjects to higher doses (10–1,000 cfu), and overt disease was observed within 3–5 days, depending upon the challenge dose. In both studies, subjects exhibited similar symptoms, such as abrupt onset of fever, headache, malaise, marked myalgia, chest tightness, and a nonproductive cough.

Subsequently, under the title Operation Whitecoat, researchers at Ft. Detrick, MD conducted 40 clinical studies of tularemia from 1958 to 1968 (16). In these studies, all human volunteer subjects were males on active duty in the Army (primarily members of Seventh Day Adventist Church) and were studied for their responses upon vaccination with a tularemia vaccine (LVS) and/or exposure to *Francisella tularensis* (called *Pasteurella tularensis* at that time in the Operation Whitecoat studies) strain Schu S4 by inhalation. The objectives of these studies varied throughout the years; evaluating the therapeutic efficacy of antibiotic regimens in alleviating the clinical symptoms associated with pneumonic tularemia, assessing the impact of disease on task performance by exposed subjects using a battery of standardized tasks, testing immunogenicity of vaccination strategies, and in some cases, determining the ability of vaccination strategies to protect against subsequent *F. tularensis* inhalation challenge.

Such human challenge studies would not be ethically feasible today. However, the rigor and depth of these archived studies led to the goal of using these data to support the regulatory approval of the animal model for future antibiotic testing. Therefore, in collaboration with clinical staff at USAMRIID, these archived data were accessed and reviewed retrospectively. To provide a better understanding of pneumonic tularemia in humans, the data in this report were limited to study arms of non-vaccinated subjects who were not treated with antibiotics until they developed symptoms associated with pneumonic tularemia. Further analysis was also performed to evaluate the efficacy of antibiotics and dosing regimens in treatment of pneumonic tularemia in humans. Thus, this report delineates the early signs and symptoms or natural history of pneumonic tularemia in a unique cohort of controlled human clinical studies and demonstrates the efficacy of tetracycline and streptomycin in treatment of disease in humans.

## Materials and Methods

### Overview of Operation Whitecoat

Approximately 2,300 men who entered military service as volunteers and were classified as conscientious objectors participated in Operation Whitecoat. Most were Seventh Day Adventists, stationed at Fort Detrick, Maryland, and were assigned duties in support of the medical research efforts such as technicians in research laboratories, hospital corpsmen, or predominantly performed administrative tasks (16). Human volunteer studies were designed by investigators in the US Army Medical Unit [now US Army Medical Research Institute for Infectious Diseases (USAMRIID)] and, after protocol review, volunteers for clinical research studies were identified, briefed, and provided informed consent documents for voluntary participation in any planned studies. Clinical staff closely monitored enrolled subjects and compiled extensive medical records and data sets for each subject.

### Data Collection and Analysis

For this retrospective analysis, USAMRIID staff extensively reviewed the existing medical records of the subjects that were enrolled in the tularemia clinical studies at Ft. Detrick. Based upon each protocol and the review of the medical records, electronic data files were structured and USAMRIID staff recorded data extracted from the paper and notebook-based medical records. Data entry personnel were instructed to enter data as they saw it in the medical records. All data entered in the database was verified via electronic queries to ensure the database matched the source documentation. All discordant data was resolved through a manual query process.

Upon completion of the data collection from each protocol, USAMRIID sent the data dictionary for each protocol along with data formatted as both csv (comma separated values) and SAS (Statistical Analysis Software) files to the National Institute of Allergy and Infectious Diseases (NIAID). NIAID worked with The Emmes Corporation (Emmes) to analyze the data and generate tables and listings that documented the clinical course of pneumonic tularemia following inhalational challenge. Emmes also flagged inconsistent data and sent queries to USAMRIID as needed.

### Selection of Subject Population

This report summarizes data from 13 challenge protocols that included a study arm where subjects were not vaccinated prior to inhalational challenge and were treated with antibiotics only after the observed onset of signs and symptoms of pneumonic tularemia. The subjects assigned to these arms in each protocol are the focus of this review, as the data collected on them following challenge provide a unique opportunity to understand the initial clinical symptoms of pneumonic tularemia in humans. The clinical presentation of pneumonic tularemia that prompted the initiation of treatment was made via the judgement of participating clinicians and was not clearly defined in the study protocols.

Across all 13 protocols, 117 subjects were considered for inclusion in the analysis. This subject population of enlisted men ranged in age from 19 to 26 years (mean 23.0) at the time of exposure. The majority of subjects were Caucasian, accounting for 94% (110/117) of the population. For detailed demographic information, refer to Supplementary Table 1.

Each of these subjects was exposed by inhalation to a specific target dose of *F. tularensis* Schu S4 and observed for symptoms associated with pneumonic tularemia. The Adverse Events (AEs) and vital signs recorded post challenge were reviewed and summarized by Emmes and NIAID. AEs were coded using Medical Dictionary for Regulatory Affairs (MedDRA, Version 20.0) to standardize the naming of the AE within and across protocols. Refer to Table 1 for a summary of the study design of each of these protocols.

**Table 1:** Protocol summaries^†^.

**Protocol number**	**Number of naïve non-vaccinated subjects**	**Target challenge dose**	**Treatment regimens**
FY 61-07	8	~200 viable *P. tularensis* (Day 0)	• 8 subjects: Treated at onset of clinical tularemia ◦ All subjects treated with 1 g streptomycin on first day, followed by 2 g streptomycin for 2 days, then either 1 g or 2 g streptomycin for 4–5 days. All streptomycin doses given IM
FY 62-01	8	~200 or ~700 viable *P. tularensis* (Day 0)	• 8 subjects: Treated at onset of clinical tularemia ◦ 2 g streptomycin, 1 g IM twice daily, for 7 days
FY 63-06	8	~2,500 or ~25,000 viable *P. tularensis* (Day 0)	• 14 vaccinees: Treated at onset of clinical tularemia • 8 non-vaccinated, control subjects: Treated at onset of clinical tularemia ◦ 2 g streptomycin, 1 g bid, either oral or IM, for 7 days
FY 64-06	6	~25,000 viable *P. tularensis* (Day 0)	• 6 subjects: Treated at onset of clinical tularemia ◦ 4 g of tetracycline on first day followed by 2 g tetracycline for the next 9 days OR ◦ 4 g of tetracycline on first day, followed by 2 g tetracycline the next 4 days, no treatment next 3 days, 2 g tetracycline the next 5 days, no treatment next 3 days, and 2 g tetracycline last 5 days • 8 subjects: receive 0.5 g tetracycline half hour before breakfast and half hour before dinner on odd days between Day 1 and Day 19, inclusively • 8 subjects: receive 0.5 g tetracycline half hour before breakfast and half hour before dinner for 42 days, starting Day 1
FY64-12	16	~25,000 viable *P. tularensis* (Day 0)	• 8 subjects: Treated at onset of clinical tularemia, 4 g tetracycline on first day followed by 2 g for next 4 days. Subjects receive 5-day course of 2 g tetracycline per day as deemed necessary. Limit of three 5 day courses may be prescribed; if relapse occurs after tetracycline treatment, subject treated with streptomycin, 1 g IM every 12 h for 14 doses • 8 subjects: Treated at onset of clinical tularemia: 4 g tetracycline on first day, followed by 2 g for next 4 days, no treatment for next 3 days, 2 g tetracycline for next 5 days, no treatment for next 3 days, and 2 g for next 5 days; if relapse occurs after tetracycline treatment, subject treated with streptomycin, 1 g IM every 12 h for 14 doses • 8 subjects: 0.5 g tetracycline orally BID for 28 days starting on Day 1; if relapse occurs after tetracycline treatment, subject treated with streptomycin 1 g IM every 12 h for 14 doses
FY 64-14	7	~2500 viable *P. tularensis* (Day 0)	• 7 subjects: Treated at onset of clinical tularemia ◦ 2 g streptomycin, orally, 1 g every 12 h for 7 days
FY64-15	8	~25,000 viable *P. tularensis* (Day 0)	• 4 subjects: Treated at onset of clinical tularemia. 4 g of tetracycline on first day followed by 2 g for 14 more days; if relapse occurs after tetracycline treatment, subject treated with streptomycin, 1 g IM every 12 h for 14 doses • 4 subjects: Treated at onset of clinical tularemia. 4 g of tetracycline on first day followed by 2 g for 20 more days; if relapse occurs after tetracycline treatment, subject is treated with streptomycin 1 g IM every 12 h for 14 doses • 4 subjects: 0.5 g tetracycline orally BID for 14 days starting on Day 1; if relapse occurs after tetracycline treatment, subject treated with streptomycin 1 g IM every 12 h for 14 doses
FY65-05	16	~25,000 viable *P. tularensis* (Day 0)	• 8 subjects: treated at onset of clinical tularemia. 4 g tetracycline on the first day followed by 2 g a day for the next 9 days (all in divided doses); if relapse occurs after tetracycline treatment, subject treated with streptomycin 1 g IM every 12 h for 14 doses • 8 subjects: treated at onset of clinical tularemia receive 4 g tetracycline for the first day followed by 2 g a day for the next 14 days (all in divided doses); if relapse occurs after tetracycline treatment, subject treated with streptomycin 1 g IM every 12 h for 14 doses • 6 subjects: 0.5 g tetracycline orally BID for 14 days, starting 24 h after exposure; if relapse occurs after tetracycline treatment, subject treated with streptomycin 1 g IM every 12 h for 14 doses
FY 65-13	6	~2500 or ~25,000 viable *P. tularensis* (Day 0)	• 16 vaccinees: treated at onset of clinical tularemia • 6 non-vaccinated, control subjects: Treated at onset of clinical tularemia ◦ 2 g streptomycin, 1 g IM twice daily for 7 days
FY66-01	8	~25,000 viable *P. tularensis* (Day 0)	• 8 subjects: treated at onset of clinical tularemia: 4 g tetracycline for the first day followed by 2 g a day for the next 14 days (all in divided doses); if relapse occurs, subject treated with streptomycin, 1 g IM every 12 h for 14 doses • 8 subjects: 1 g of tetracycline orally BID for 14 days beginning 24 h after exposure; if relapse occurs, subject treated with streptomycin 1 g IM every 12 h for 14 doses
FY66-13	16^*^	~25,000 viable *P. tularensis* (Day 0)	• 8 subjects (6-day adaptation, control and training period prior to exposure), treated at onset of clinical tularemia. 4 g tetracycline in 4 doses on the first day, followed by 2 g a day in 4 doses for 14 days; if relapse occurs, subject treated with streptomycin, 1 g IM every 12 h for 14 doses • 8 subjects (3-day adaptation, control and training period prior to exposure) treated at onset of clinical tularemia. 1 g tetracycline, orally, BID for 14 days; if relapse occurs, subject treated with streptomycin, 1 g IM every 12 h for 14 doses
FY67-01	8	~25,000 viable *P. tularensis* (Day 0)	• 8 subjects treated at onset of clinical tularemia with streptomycin 1 g IM twice a day for 7 days.
FY 68-04A	4	~2500 or ~25,000 viable *P. tularensis* (Day 0)	• 16 vaccinees: Treated at onset of clinical tularemia • 4 non-vaccinated, control subjects: Treated at onset of clinical tularemia ◦ 2 g streptomycin, 1 g IM twice daily, for 7 days

* *Note that for this group of subjects, only 14 subjects were included in the analyses. Two subjects (66-13-011, 66-13-017) did not exhibit any spikes in body temperature and were not treated with tetracycline and were thus excluded*.

† *Note that some of the Treatment Regimens presented herein (albeit not specifically identified) and a brief discussion of antibiotic efficacy have been previously published (17)*.

### Inhalational Exposure to *F. tularensis* Schu S4

Study subjects were exposed to aerosolized *F. tularensis* by mask, using a modified Henderson apparatus (18). Inspiration was through the nose and expiration was through the mouth, where a dry test meter recorded expiratory volume. The mask was connected to the aerosol apparatus through a by-pass valve that allowed for breathing filtered room air before and after the exposure but also allowed termination of exposure by either the volunteer or the operator at any time, which did not appear to occur for any of the subjects selected for analysis. Bacterial concentration (cfu/ml) in the aerosol was determined by sampling the aerosol with all-glass impingers (AGI) followed by plating and enumeration of colony growth to determine the *F. tularensis* exposure dose.

The exact time of exposure was not defined in all protocols and only recorded in three protocols (64-06 @0830 h, 66-13 @1100 h, and 67-01 @0945 h). It was evident from review of the protocols that exposure was to occur in the morning. Therefore, a standard exposure time of 9:00 a.m. was used for all subjects.

### Clinical Parameters Collected: Vital Signs

**Figure 1:**
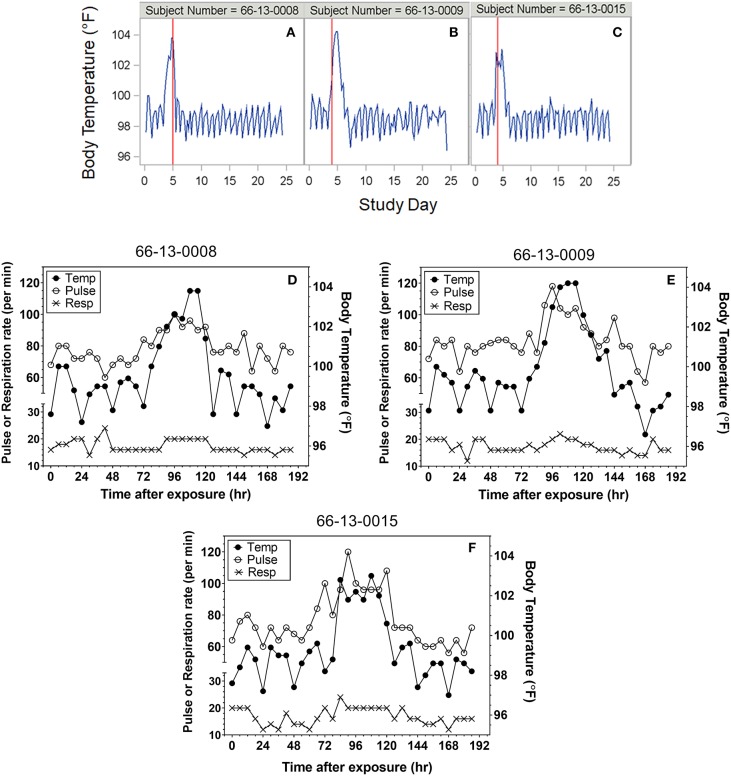
Representative plots of vital signs for subjects exposed to 25,000 cfu. **(A–C)** Representative temperature plots derived from body temperature (in °F) listings over the entire course of the study. The red vertical line represents the study day (@ 000 h) where antibiotic was administered. **(D–F)** Overlay plots for the same subjects in which body temperature changes (closed circles, in °F) are plotted with pulse rate (open circles) and respiration rate (X) over the first eight study days after exposure.

Heart rate, temperature, and respiratory rate were measured and documented every 6 h following exposure for most protocols. Although not specified in every protocol, where it was indicated temperatures were taken rectally. Representative plots of the time courses for body temperature in subjects treated with antibiotic are shown in Figures 1A–C. For reporting purposes, the day of exposure was set as Day 0, with subsequent solar days reported as Day 1, Day 2, etc. A majority of subjects exhibited varying degrees of tachypnea and tachycardia coincident with the onset of other clinical symptoms. However, neither vital sign was a reliable indicator of disease onset compared to temperature. Representative overlay plots of the time courses for temperature, respiration rate and heart rate are shown in Figures 1D–F. The data are from the same subjects shown in Figures 1A–C and are focused upon the changes up to Study Day 8.

### Objective Definition of Fever Onset

Although all 13 protocols included in this report collected body temperatures approximately every 6 h on all the subjects, none defined fever. Four of the protocols (64-12, 64-15, 65-05, and 66-01) recorded fever as an Adverse Event (AE) (coded as pyrexia), but there was no description of temperature changes that triggered the coding. Therefore, in order to standardize the definition of fever, it was decided to utilize the temperature recordings. As described above, to establish a time to fever in hours the time of exposure was set for 9:00 a.m. for all subjects.

Upon review of the data and to limit the effect of diurnal fluctuations in temperature, an objective definition of fever was set to be two consecutive temperature readings of 100°F or greater. As described below, this definition was consistent with the initiation of treatment and the presence of other symptoms of pneumonic tularemia in the majority of subjects.

### Clinical Parameters Collected: Laboratory Parameters and Chest X-Ray

Many laboratory/clinical chemistry parameters were measured but were not consistently available for all studies. The parameters reported in these studies included: complete blood count (CBC) with differential, albumin/globulin ratio, albumin, alkaline phosphatase, aspartate aminotransferase, alanine aminotransferase, bilirubin (total and direct), glucose, blood urea nitrogen, total protein, sedimentation rate, C-reactive protein (CRP), lactate dehydrogenase, and cardiolipin antibodies. In addition, blood, stool and pharyngeal washes were cultured to determine bacterial load. Finally, chest x-rays and urinalysis were performed.

Although the parameters measured were similar among the protocols, the time-points of data collection were rarely consistent with respect to time interval post-challenge. Furthermore, most studies did not have baseline measures and many parameters were measured well after initiation of antibiotic treatment, limiting their utility as measures of disease progression. Therefore, no trends in the data or changes in values related to challenge that would assist in characterizing the clinical presentation of the disease following exposure could be determined. Thus, it was concluded that these parameters would not contribute to the assessment of pneumonic tularemia in this population and these data are not included in this report.

## Results

### Exposure Levels

Supplementary Table 2 presents summary statistics for exposure levels to *F. tularensis*. Most subjects (72.6%; 85/117) were exposed to a target dose of 25,000 cfu, but smaller numbers were exposed to lower doses [200 cfu (12 subjects), 700 cfu (4 subjects), and 2,500 cfu (16 subjects)]. While those exposed to higher doses all experienced fever, only 50% of those exposed to 200 cfu developed fever, and those subjects were not treated with antibiotics and only one of them developed serum antibodies as detected by hemagglutination (19).

### Determination of Disease Onset: Time to Fever

**Figure 2:**
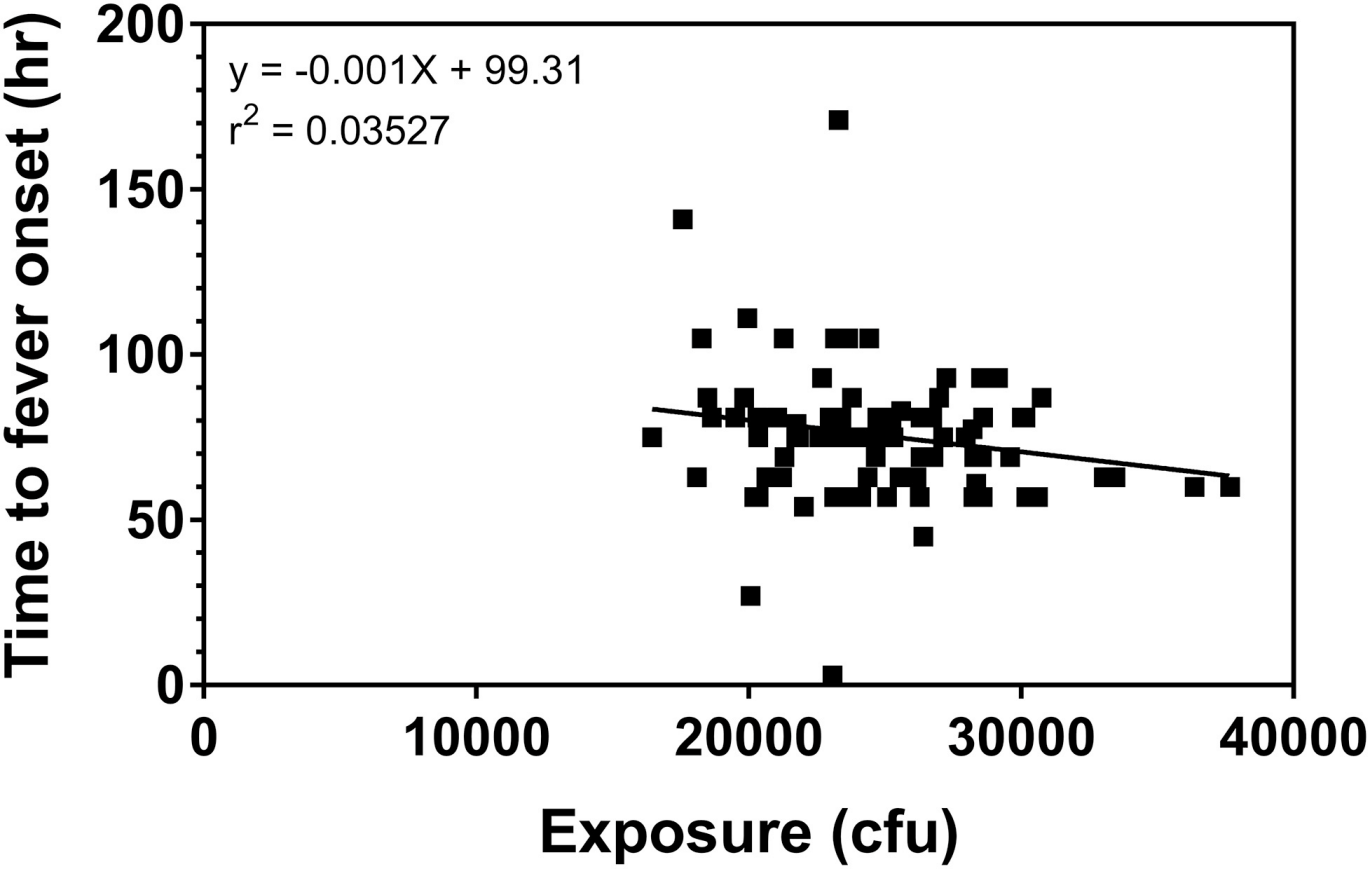
Relationship of challenge dose to time of fever onset. Time to fever onset is calculated for each subject from temperature listings (as described in the Methods) as the first time point in which the subject exhibited 2 consecutive readings ≥100°F. The data represent the calculated exposure (as described in the methods) to *F. tularensis* Schu S4 in those subjects exposed to a target exposure = 25,000 cfu.

Using the fever criteria defined in the Methods, time to fever after exposure to *F. tularensis* was determined using the temperature listings. The mean time to fever for each protocol and overall is presented in Table 2. Focusing upon the population exposed to 25,000 cfu, the mean time to fever (± standard deviation) was 74.4 ± 20.9 h after exposure to *F. tularensis*. Variability across and within protocols was observed, however measuring time to fever in hours allows for a definitive time period where most subjects exhibited fever. The variability in the mean between protocols did not exceed 29 h, providing a fairly small window of time when fever occurred for the majority of subjects. When assessing the relationship of time to fever with exposed dose in the group exposed to a target dose of 25,000 cfu, it is clear that time to fever onset does not vary substantially across that higher range of doses (*r*^2^ = 0.035) (Figure 2).

**Table 2:** Time to fever by protocol and overall^a^.

	**Protocol**																	
	**61-07**	**62-01**		**63-06**		**64-06**	**64-12**	**64-14**	**64-15**	**65-05**		**65-13**	**66-01**	**66-13**	**67-01**	**68-04A**		**Overall**
Target exposure (cfu)	200	200	700	2,500	25,000	25,000	25,000	2,500	25,000	25,000	2,500	25,000	25,000	25,000	25,000	2,500	25,000	
Number of subjects with a fever^*a*^	4	2	4	4	4	6	16	7	8	16	3	3	8	14	8	2	2	111
Mean time to fever (hour)	159.0	198.0	162.0	85.5	79.5	68.1	85.5	114.4	81.8	77.6	109.0	58.0	56.3	68.6	85.5	107.0	81.0	88.1
SD	38.88	140.01	76.76	19.21	10.25	9.53	33.33	38.71	16.80	12.58	34.12	1.73	5.26	21.24	12.73	0.00	2.83	40.17
Median time to fever (hour)	162	198	135	93	78	66	81	99	81	75	99	57	57	72	81	107	81	81
Min	111	99	105	57	69	57	27	93	63	57	81	57	45	3	75	107	79	3
Max	201	297	273	99	93	81	171	201	105	105	147	60	63	87	105	107	83	297

a *Time to fever was determined for each subject (as defined in section Data Collection and Analysis) using the first time point of two consecutive temperatures of 100°F or greater and assumes a 9 a.m. challenge time on Day 0. Subjects who did not present with a fever are not included in these statistics. 6 subjects didn't have fever*.

Subjects at the extreme minimum and maximum times to fever were further assessed. Subject (66-13-0008) exhibited two consecutive temperature readings of exactly 100°F on the day of exposure suggesting a time to fever of 3 h. The temperature immediately returned to baseline, followed by increased body temperature (at 75 h post-exposure) and clinical symptoms of headache, malaise and nausea and vomiting on Day 4 post exposure, which would be consistent with what was observed with the majority of subjects exposed to 25,000 cfu target dose. Treatment with tetracycline was initiated on Day 5 post exposure. If the temperature elevation on the day of exposure was not considered, the calculated time to fever would have been 75 h, a time close to the mean time to fever for all subjects. Nonetheless, the time to fever used for the analysis for this subject was determined by the definition and did not reflect the full clinical presentation and response by the clinical staff.

Another subject (64-12-0021) had a single temperature spike of 102.2°F on Day 4 post exposure but did not present with associated symptoms. An AE of pyrexia was recorded for Day 4 but he was not treated with tetracycline at this time. On Day 7 post exposure this subject again experienced an elevated temperature that was associated with malaise and substernal chest pain. The calculated time to fever, using our definition, was 171 h and tetracycline therapy was initiated on Day 8 post exposure.

In yet another subject (64-12-0023), the definition of fever was not consistent with the full clinical presentation of the subject and the study clinician's evaluation. By definition, this subject developed fever at 27 h, but pyrexia was reported by the study clinician as an AE on Day 4 after exposure. However, the start date of the AE did not correspond to the day when his temperature again reached above 100°F (Day 5) and he was treated with tetracycline. Similarly, his other symptoms of headache, substernal chest pain and insomnia also started 5 days post exposure.

Due to the retrospective nature of this analysis, clarification of the events is not possible. Nevertheless, exclusion of the above three subjects did not have a significant impact on the overall mean, so their data were kept in the analysis.

### Reporting of Adverse Events

As is standard in the presentation of Adverse Events (AEs), Emmes used MedDRA to code the AE descriptions reported on each of the protocols. There were up to 98 different AEs reported across all protocols by MedDRA preferred terms, but this report focused upon those events having an incidence of 20% or more in the subject population. All subjects in this data set, with the exception of the 4 subjects exposed to 200 cfu described above, experienced a fever, though it was recorded as an AE only in some protocols. A summary of the different AEs and their mean time to onset are shown in Supplementary Table 3.

**Figure 3:**
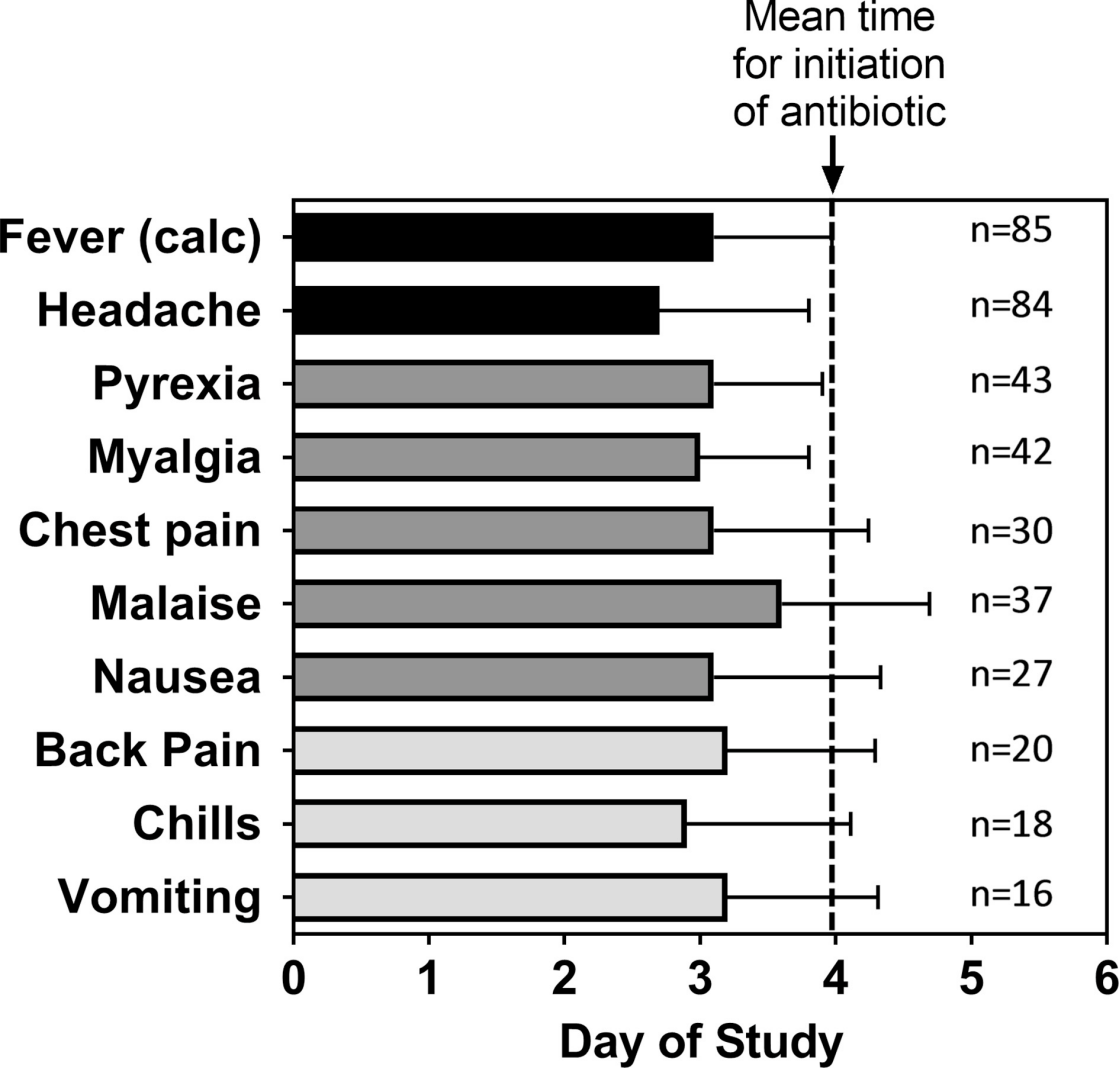
Mean time for adverse events after inhalational exposure to *F. tularensis* Schu S4. For the indicated number (*n*) of subjects that reported the given Adverse Event, the mean study day (± SD) when each of the adverse events were first reported was calculated. The data are compared to the calculated time of fever onset [Fever (calc)] as shown in Table 2 and the mean study day when antibiotic treatment was initiated (dashed line). The data represent only subjects exposed to a target exposure of 25,000 cfu.

Focusing upon those subjects exposed to a target dose of 25,000 cfu, headache was reported by 99% of all subjects and accounted for one of the earliest symptoms reported by the subjects. There were 50.6% of the subjects with fever reported as an AE (coded as Pyrexia). Nearly half of the subjects reported myalgia (49%), malaise (44%), and chest pain (35%—which included substernal, and retrosternal). Many subjects reported nausea (32%), back pain (24%), chills (21%), and vomiting (20%). In general, the timing of the AEs in these subjects was consistent with the onset of disease (Figure 3) and because antibiotic treatment was initiated typically within 1 day of disease onset, advanced disease sequelae (i.e., pulmonary symptoms) were not observed in these subjects. Nevertheless, the data suggest that certain AEs, such as headache or chills, were reported earlier than others such as malaise or vomiting.

### Effect of Exposure Dose on Disease Onset

**Figure 4:**
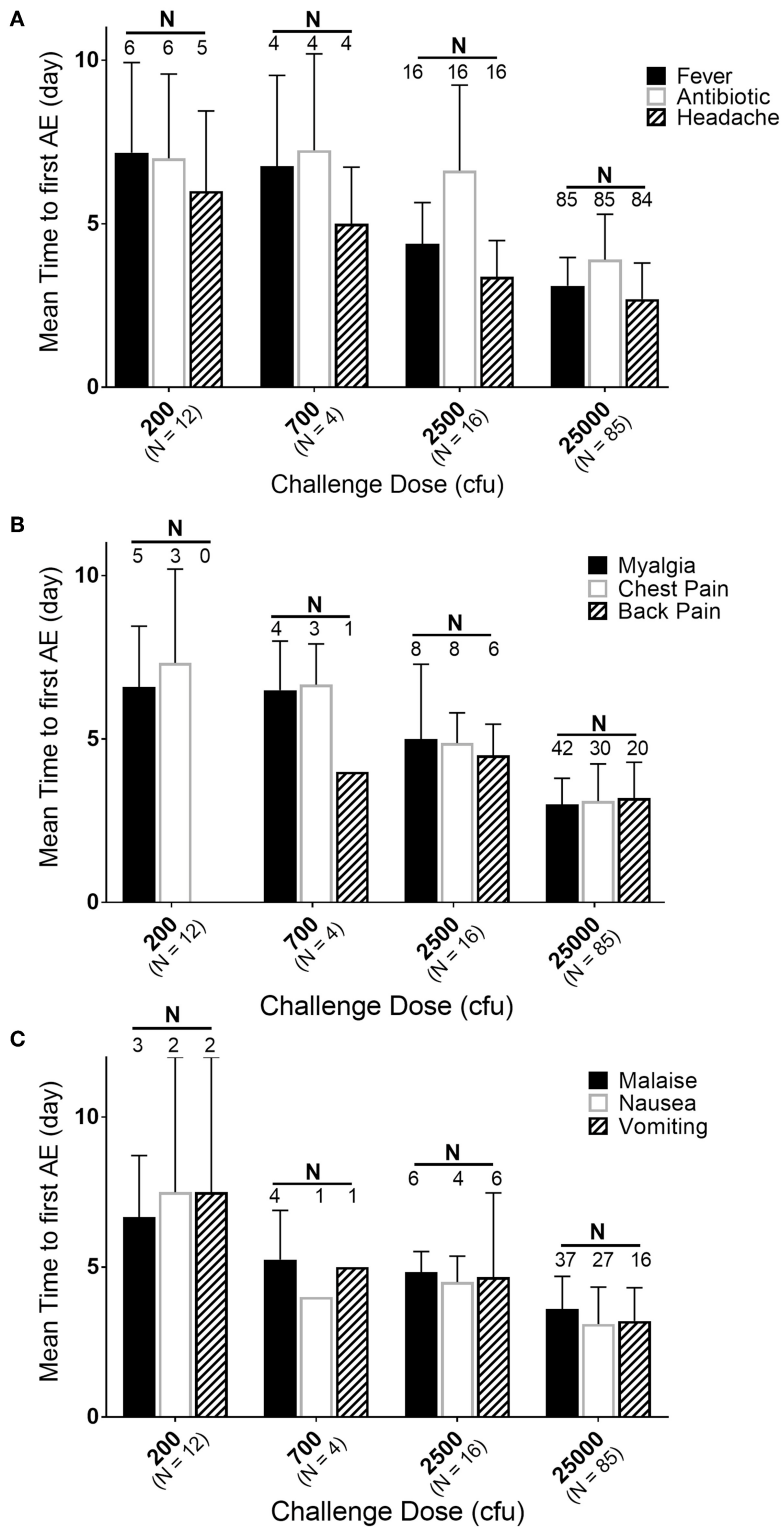
Relationship of mean time to adverse event onset to exposure dose. Mean day (± SD) of first reporting of Adverse Events upon challenge with indicated doses of *F. tularensis* Schu S4. **(A)** Represents those in the majority or all of subjects, **(B)** represents AEs present in about half of subjects and **(C)** those in about 30% of subjects. Number of subjects exposed are indicated with dose, while the number of subjects that had a particular AE are labeled above the respective bars. Fever is calculated in hours and back-calculated to days, while other AEs and antibiotic administration are only reported by day of study. Note that only 50% of subjects exposed to 200 cfu experienced fever. Any of those exposed to 200 cfu that did not get fever, also did not get treated with antibiotic and only one developed antibodies by agglutination microtiter.

While the majority of the subjects were exposed to 25,000 cfu, there were groups of subjects exposed to lower doses in the same or different studies. If the timing and effects of the lower exposure doses (200, 700, and 2,500 cfu) are compared to the responses in the subjects exposed to 25,000 cfu, a dose-response relationship can be observed (Figure 4). Supplementary Table 3 presents a summary by exposure dose with time to fever (by the definition in the Methods, but listed in days) after *F. tularensis* exposure, the time to the first reporting of symptoms (restricted to symptoms experienced by 20% or more of the subjects) and time to antibiotic therapy. As discussed above, the majority of the AEs in subjects exposed to 25,000 cfu were reported on Day 3. However, as the exposure doses decreased from 2,500 to 200 cfu, the study day of onset of AEs extended from Day 4 up to Day 7. For example, the time to onset of fever increased from 75.5 h (@ 25,000 cfu), to 105.3 h (@ 2,500 cfu) and to 162 h (@ 700 cfu) and 172 h (@ 200 cfu). Interestingly, the total percentage of subjects at each exposure dose that reported the different AEs remained relatively similar across the different doses, suggesting that while disease onset took longer, the typical presentation of disease was similar across the different exposures.

### Efficacy of Tetracycline in Subjects Exposed to Aerosolized *F. tularensis* Schu S4

Antibiotic was administered upon presentation with clinical tularemia, as determined by clinical staff. For the current analysis of tetracycline efficacy, only subjects exposed to 25,000 cfu *F. tularensis* Schu S4 were examined, since all subjects with lower exposures were treated with streptomycin (which was uniformly efficacious). Furthermore, the eight (8) subjects in Study 67-01 were treated with streptomycin and were excluded. Thus, using the definition of fever described in the Methods, 68 subjects experienced an acute onset of fever and were treated with tetracycline after disease onset.

Although not defined in the associated protocols, the initiation of antibiotic treatment was often, but not always, within 1 day after onset of fever (as seen in Figure 3). Across the studies, several different dosing schedules for tetracycline were used (summarized in Table 1). The majority of subjects (63 of 68; 92.6%) analyzed were treated daily for 9 days to 24 days, although 4 of 68 (5.9%) subjects (only in Studies FY64-06 and FY64-12) were treated in a staggered fashion (e.g., 5 days on, 3 days off, 5 days on, etc.). In all cases, tetracycline was administered at either 1 or 2 g total per day, typically q.i.d., although in some cases (22 of 68; 32.4%) there was a loading dose of 4 g on the first day of administration. Table 1 summarizes the dosing schedules for all the subjects analyzed and a summary of the treatments and responses of each of the subjects is listed in Supplementary Table 4.

Subjects were monitored for 4–5 weeks after aerosol challenge and the relevant measures recorded for the subjects were body temperature and the occurrence of AEs (e.g., headache, chest pain, malaise). Because subjects were treated soon after onset of disease, more advanced pulmonary symptoms of pneumonic tularemia were not observed. Representative temperature plots for subjects treated with efficacious doses of tetracycline are shown in Figure 1. Among those studied, seven (7) subjects (64-06-0014, 64-06-0005, 65-05-0004, 65-05-0008, 64-06-0022, 66-01-0003, 66-01-0011) were deemed to have “relapsed” after termination of the first course of tetracycline. For the purposes of this report, relapse was defined as requiring a second round of antibiotic treatment; streptomycin, 1 g i.m. b.i.d. for 7 days, which was effective in clearing the infection. Relapse was typically associated with re-appearance of AEs and another spike in body temperature consistent with fever, as defined in the Methods. One subject (66-13-0012) had a severe reaction to tetracycline treatment after 2 days of treatment and was immediately treated with streptomycin; this subject was excluded from this analysis.

To examine the efficacy of tetracycline treatment in these subjects with respect to disease relapse, the analysis focused upon those subjects treated continuously with tetracycline. Table 3 summarizes the 63 subjects who were treated daily with tetracycline, separating them based upon treatment duration less than or greater than or equal to 14 days and daily dosage (1 or 2 g tetracycline per day). Treatment for <14 days resulted in significant rate of relapse, while treatment with the higher dose (2 vs. 1 g per day) for at least 14 days significantly decreased the occurrence of relapse.

**Table 3:** Percent relapse (streptomycin follow-up treatment) in operation whitecoat subjects exposed to aerosolized *F. tularensis* Schu S4.

**Duration of treatment**	**1 g per day (0.25 g q.i.d)**	**2 g per day (0.5 g q.i.d or 1 g b.i.d)**	**Totals**
<14 days	NA	5 of 11 subjects (45.5%)	5 of 11 subjects (45.5%)
≥14 days	2 of 8 subjects (25%)	0 of 44 subjects (0%)^*^^†^	2 of 52 subjects (3.8%)^*^
Totals	2 of 8 subjects (25%)	5 of 55 subjects (9.1%)	7 of 63 subjects (11.1%)

* *Significantly different from subjects treated <14 days (p < 0.001)*.

† *Significantly different from subjects treated with 1 g per day (p = 0.02)*.

Aside from those that had disease “relapse,” other subjects also exhibited evidence of transient increases in body temperature and re-appearance of AEs after termination of tetracycline treatment (Table 4), although they were not necessarily defined as relapsed or treated with streptomycin by clinical staff. Subjects with reappearance of AEs were defined as those with an AE start date >9 days post-antibiotic start date, including any of those defined as relapsed above. Nine days post-antibiotic start was chosen since that was the minimum duration of antibiotic treatment and the goal was to identify situations in which treatment was ineffective and allowed disease symptoms to reappear. In all the cases that were not treated with streptomycin, disease symptoms resolved themselves without additional treatment, suggesting active host immune responses cleared any residual infection.

**Table 4:** Percent of subjects showing re-appearance of more than 1 adverse event after termination of tetracycline treatment in operation whitecoat subjects exposed to aerosolized *F. tularensis* Schu S4.

**Duration of treatment**	**1 g per day (0.25 g q.i.d)**	**2 g per day (0.5 g q.i.d or 1 g b.i.d)**	**Totals**
<14 days	NA	6 of 11 subjects (54.5%)	6 of 11 subjects (54.5%)
≥14 days	1 of 8 subjects (12.5%)	8 of 44 subjects (18.2%)^*^	9 of 52 subjects (17.3%)^*^
Totals	1 of 8 subjects (12.5%)	14 of 55 subjects (25.5%)	15 of 63 subjects (23.8%)

* *Significantly different from subjects treated <14 days (p < 0.05)*.

Similar to the data for relapse, there was a significant difference in antibiotic efficacy depending upon treatment schedule when reappearance of AEs was considered. While there was no apparent difference in treatment with 1 vs. 2 g of tetracycline, there was significantly better efficacy when tetracycline treatment was extended to at least 14 days (Table 4).

Note that the protocol summary for FY64-12 (Table 1) indicated that at least 8 subjects were treated with 2 g tetracycline in an “interrupted” course (on and off) after onset of clinical tularemia. This regimen was also suggested by the associated documents (abstract, progress report—data not shown). However, based upon the data files used that described drug treatments, 64-12-0011 was the only subject treated as such. Given the unusually high incidence of spikes in body temperature and recurrent AEs in FY64-12 subjects as compared to those in the other protocols, it seems likely that more subjects were treated in an interrupted schedule with tetracycline. Therefore, the reported incidence of additional temperature spikes or recurrence of AEs after the end of tetracycline treatment is probably somewhat skewed by this possible discrepancy.

## Discussion

The retrospective analysis performed on data from the subjects challenged with *F. tularensis* by inhalation has provided a unique opportunity to assess very useful information regarding the course of pneumonic tularemia in humans. The depth and quality of data from these historical studies are not commensurate with that generated by clinical studies today, making comparison to recent data difficult. However, most data from clinical reports within the past 2–3 decades lack the granularity of time, dose, and route of exposure. Because the subjects in this analysis were challenged with a known agent dose and closely monitored by clinicians, this study provides important details pertaining to the early events following inhalational exposure to a pathogen that is considered to be a potential biologic weapon.

Due to the fact that the protocols lacked definition of fever and signs and symptoms of pneumonic tularemia, an effort was made to characterize not only time to fever but symptomatology present around the time of fever. The initiation of antibiotic treatment was also considered, as this could provide an indication of when clinicians monitoring a subject believed the subject was demonstrating characteristics of active infection. One factor to note is that the time to fever could be calculated in hours and thereby determined more specifically than time to antibiotic therapy or time to AE, as all vital signs were noted by time and date. Only the date was recorded for initiation of antibiotic therapy and for the onset of an AE, therefore the presentation is less precisely recorded. It is worthy to note that the overall mean time to reported pyrexia (3.1 ± 0.8 days) in the subset of studies where it was reported was consistent with the overall mean of time to fever using the definition in this analysis (3.2 ± 0.9 days).

In order to characterize the natural history of pneumonic tularemia in these human subjects, the inclusion of symptoms occurring after initiation of antibiotic therapy or were indicative of relapse or unrelated illness were minimized in this analysis. Thus, those data analyses excluded all symptoms that were reported either prior to challenge or more than 1 day after treatment initiation. The results demonstrate that the vast majority of subjects experienced symptoms associated with pneumonic tularemia either the same day or within a day of having a fever (as defined by our criteria in the Methods). Headache was reported by essentially all the subjects and was closely aligned with the onset of fever. Similarly, although reported by fewer subjects (or in a subset of the studies), symptoms such as body pain (myalgia, chest pain, back pain) as well as nausea and vomiting were also associated with the development of fever. Because antibiotic therapy was initiated within a day or two after disease onset, symptoms associated with prolonged disease in humans (20–22), such as lung involvement (e.g., pneumonia, cough, wheezing) were not reported.

In most subjects exposed to high doses of the pathogen (25,000 cfu), the clinical presentation of fever and associated symptoms occurred by Day 3 or Day 4, with a mean time to fever of 74.4 h. Interestingly, there appeared to be a dose-response relationship in terms of the onset of symptoms, with lower aerosol exposures leading to a longer lag between challenge and apparent infection. Furthermore, even at the lower exposures, the onset of symptoms was still associated temporally with the onset of fever. Thus, fever seems to be one of the earliest indicators of disease in these subjects with other major symptoms of disease/AEs reported within a day of the onset of fever. A review of other clinical parameters collected in these studies did not reveal any trends or indicators of disease.

As opposed to the natural history of progression of pneumonic tularemia in the human subjects, analysis of the efficacy of tetracycline focused upon the time after initiation of antibiotic treatment. In all the studies in this report, streptomycin treatment was the treatment of choice to eliminate infection, as 1 g b.i.d. for 7 days was 100% efficacious. However, most of the studies reported herein had study arms that tested different dosing and schedules for tetracycline as an alternative treatment. When antibiotic treatment was initiated upon onset of clinical tularemia, drug administration typically occurred within 1 day of establishment of disease as defined by fever onset. Of the 68 subjects treated with tetracycline after disease onset in the study group, there were only 9 subjects for whom antibiotic therapy was not initiated either the same day as the onset of fever or the next day. Two of these subjects (66-13-0008 and 64-12-0023) were discussed above in the context of definition of fever onset. In these two subjects, antibiotic therapy was initiated when temperatures consistent with fever were again subsequently present and symptoms (AE) of tularemia were reported. Subject 65-05-001 developed fever 2.4 days post challenge and received antibiotic therapy on day 4 post challenge. Although not within 1 day of fever onset, the clinical presentation and response by the clinicians appears to be appropriate in this subject. Upon review of the other six subjects (64-12-0013, 64-12-0022, 64-12-0024, 64-15-0009, 66-01-0001, and 66-01-0007), there is no explanation why antibiotic therapy was not initiated until 3–4 days post onset of fever and the presence of symptoms.

While the majority of subjects were treated with antibiotic daily, there were study arms in which intermittent dosing schedules were tested. However, these were previously found to be less effective (17) and are not consistent with the recommended continuous treatment regimen for tetracycline (23–25). Therefore, this retrospective analysis focused primarily upon those subjects (63 of 68 subjects treated with tetracycline as the primary treatment) treated daily with tetracycline after disease onset at different doses and for varying durations.

Indicators of antibiotic efficacy available for analysis were body temperature and reporting of AEs. In a subset of the tetracycline treated subjects (8 of 63 subjects), disease was deemed to have relapsed and the subjects received the streptomycin regimen and subsequently cleared the infection. The relapse was often associated with lower doses of tetracycline or shorter durations of treatment and was reflected by another spike in body temperature and/or recurrence of at least two AEs associated with clinical tularemia more than 9 days after starting antibiotic treatment. Most of the relapses occurred near the end of or within 10 days after termination of antibiotic treatment, suggesting that the bacteriostatic tetracycline was not sufficient to halt the bacterial infection under those conditions. While all the subjects in these groups were exposed to the highest exposure dose (25,000 cfu), the data suggest that longer treatment or administration of higher doses of tetracycline were efficacious in clearing infection. Stratifying subjects into groups treated with tetracycline for either 1 or 2 g/day and for treatment durations less than or greater than 14 days revealed significant differences in treatment outcomes.

The analyses of these studies conclude that an optimal treatment regimen for tetracycline would be 2 g/day for at least 14 days to minimize relapse and additional symptoms associated with pneumonic tularemia. Note that a group of subjects who exhibited a second spike of body temperature and/or AEs after antibiotic treatment were not treated with streptomycin, but still resolved disease suggesting that the immune responses induced by exposure were sufficient to clear the infection. Thus, for the bacteriostatic tetracycline a balance of antibiotic and host defenses likely contribute to clearance of infection after inhalational challenge with *F. tularensis*. This estimation of an optimal treatment regimen is consistent with that presented in an analysis of a subset of these subjects (17). From the FDA label, tetracycline dosage is typically 1 g per day, but possibly 2 g per day for severe infections (2).

Therefore, the current retrospective analysis of this unique study population of controlled human trials shows remarkable parallels with the already published human studies (14, 15, 17) in terms of disease presentation and timing. The earlier DoD-supported studies (14, 15) also had controlled exposure to low levels of Schu S4 using similar aerosol generators as in the Operation Whitecoat studies. Unfortunately, although also DoD sponsored, we did not have access to the data from these studies, but the disease progression and presentations were highly similar with comparable time of disease onset and symptoms for pneumonic tularemia as the current report, with abrupt onset of fever, headache, malaise, marked myalgia, chest tightness, and a non-productive cough.

The existence of these archived data presented an opportunity to advance our understanding of disease caused by a NIAID Category A priority pathogen in humans. The defined nature of the challenges and documentation of clinical monitoring provided an in-depth characterization of disease progression and further enhance the understanding of pneumonic tularemia in humans. Such human challenge studies are not ethically feasible today and intensive efforts are in place to establish animal models for testing of vaccines and countermeasures in the future. Thus, the data provide support to the use of the cynomolgus macaque model under the Animal Rule, since the data in this report for human disease are also comparable to the reports of pneumonic tularemia in the non-human primate model (11, 26).

## Data Availability Statement

The datasets generated for this study are available on request to the corresponding author.

## Author Contributions

PP and BF led the effort to convert archived notebook data to electronic records. HH and JM led the efforts to organize and extract relevant data from electronic records and generated summary tables and reports. MB, TG, LL, and JH established the contract with USAMRIID and the data requirements for extraction. MB and MW analyzed data, interacted with The Emmes Company to produce summary tables and reports, and wrote the manuscript.

### Conflict of Interest

The authors declare that the research was conducted in the absence of any commercial or financial relationships that could be construed as a potential conflict of interest.
