# Supplementary material for: Retrospective Analysis of Pneumonic Tularemia in Operation Whitecoat Human Subjects: Disease Progression and Tetracycline Efficacy
Source: Front Med (Lausanne). 2019 Oct 22;6:229. doi: 10.3389/fmed.2019.00229 (PMC6818494; doi:10.3389/fmed.2019.00229)
Supplement: Supplementary file 1 [file Data_Sheet_1.docx]

**SUPPLEMENTAL DATA**

**Retrospective Analysis of Pneumonic Tularemia in Operation Whitecoat Human Subjects: Disease Progression and Tetracycline Efficacy**

**Mark S Williams^1^, Tina Guina^1^, Marianne R Baker^1^, Judith A Hewitt^1^, Lynda Lanning^2^, Heather Hill^3^, Jeanine M. May^3^, Beverly Fogtman^4^ and Phillip R Pittman^4^**

^1^Office of Biodefense Research Resources and Translational Research, ^2^Office of Regulatory Affairs, Division of Microbiology and Infectious Diseases, National Institute of Allergy and Infectious Diseases, National Institutes of Health, Bethesda, MD, USA

^3^The Emmes Company, Rockville, MD, USA

^4^United States Army Medical Research Institute of Infectious Diseases, Fort Detrick, MD, USA

*** Correspondence:**Mark S Williams
mark.williams4@nih.gov

**Table 1: Demographics by Protocol and Overall**

|  | **Protocol** | | | | | | | | | | | | | |
| --- | --- | --- | --- | --- | --- | --- | --- | --- | --- | --- | --- | --- | --- | --- |
|  | **61-07** | **62-01** | **63-06** | **64-06** | **64-12** | **64-14** | **64-15** | **65-05** | **65-13** | **66-01** | **66-13** | **67-01** | **68-04A** | **Overall** |
| Sex |  |  |  |  |  |  |  |  |  |  |  |  |  |  |
| Male | 8 (100%) | 8 (100%) | 8 (100%) | 6 (100%) | 16 (100%) | 7 (100%) | 8 (100%) | 16 (100%) | 6 (100%) | 8 (100%) | 14 (100%) | 8 (100%) | 4 (100%) | 117 (100%) |
| Race |  |  |  |  |  |  |  |  |  |  |  |  |  |  |
| Asian | 0 (0%) | 0 (0%) | 0 (0%) | 0 (0%) | 0 (0%) | 0 (0%) | 1 (13%) | 0 (0%) | 0 (0%) | 0 (0%) | 1 (7%) | 0 (0%) | 0 (0%) | 2 (2%) |
| Black or African American | 0 (0%) | 1 (13%) | 1 (13%) | 0 (0%) | 1 (6%) | 0 (0%) | 1 (13%) | 1 (6%) | 0 (0%) | 0 (0%) | 0 (0%) | 0 (0%) | 0 (0%) | 5 (4%) |
| White | 8 (100%) | 7 (88%) | 7 (88%) | 6 (100%) | 15 (94%) | 7 (100%) | 6 (75%) | 15 (94%) | 6 (100%) | 8 (100%) | 13 (93%) | 8 (100%) | 4 (100%) | 110 (94%) |
| Age at *F. tularensis* exposure |  |  |  |  |  |  |  |  |  |  |  |  |  |  |
| N | 7* | 8 | 8 | 6 | 16 | 7 | 8 | 16 | 6 | 8 | 14 | 8 | 4 | 116 |
| Mean | 22.6 | 22.8 | 23.8 | 23.8 | 23.3 | 24.0 | 23.3 | 22.7 | 22.3 | 22.5 | 22.8 | 22.5 | 22.8 | 23.0 |
| SD | 2.23 | 1.98 | 1.67 | 0.75 | 2.14 | 1.00 | 1.49 | 1.49 | 0.52 | 0.76 | 0.97 | 0.53 | 1.71 | 1.50 |
| Median | 23 | 23 | 24 | 24 | 24 | 24 | 24 | 23 | 22 | 23 | 23 | 22 | 22 | 23 |
| Min | 19 | 19 | 20 | 23 | 19 | 22 | 21 | 19 | 22 | 21 | 20 | 22 | 21 | 19 |
| Max | 25 | 25 | 25 | 25 | 26 | 25 | 25 | 24 | 23 | 23 | 24 | 23 | 25 | 26 |

* Note that one subject from this protocol did not report an age or date of birth

**Table 2: Actual Exposure to *F. Tularensis***

|  | **Protocol** | | | | | | | | | | | | | | | | | |
| --- | --- | --- | --- | --- | --- | --- | --- | --- | --- | --- | --- | --- | --- | --- | --- | --- | --- | --- |
|  | **61-07** | **62-01** | | **63-06** | | **64-06** | **64-12** | **64-14** | **64-15** | **65-05** | **65-13** | | **66-01** | **66-13** | **67-01** | **68-04A** | | **Overall** |
| **Targeted Exposure (cfu)** | **200** | **200** | **700** | **2,500** | **25,000** | **25,000** | **25,000** | **2,500** | **25,000** | **25,000** | **2,500** | **25,000** | **25,000** | **25,000** | **25,000** | **2,500** | **25,000** |  |
| Actual Exposure (viable *F. tularensis)* |  |  |  |  |  |  |  |  |  |  |  |  |  |  |  |  |  |  |
| N | 8 | 4 | 4 | 4 | 4 | 6 | 16 | 7 | 8 | 16 | 3 | 3 | 8 | 14 | 8 | 2 | 2 | 117 |
| Mean | 212.5 | 200.0 | 700.0 | 2212.8 | 24607.8 | 27182.8 | 22988.7 | 1860.1 | 20264.5 | 25075.8 | 2776.3 | 31601.0 | 26723.0 | 26779.1 | 23896.1 | 2164.5 | 23674.0 | 18414.7 |
| SD | 40.09 | 0.00 | 0.00 | 227.04 | 1657.87 | 1943.72 | 3291.47 | 427.90 | 2096.65 | 3404.35 | 377.57 | 4240.65 | 5855.91 | 3669.99 | 3459.12 | 303.35 | 2719.53 | 11156.03 |
| Median | 213 | 200 | 700 | 2298 | 24506 | 27436 | 22780 | 1973 | 20074 | 25921 | 2768 | 30187 | 25672 | 26730 | 23563 | 2165 | 23674 | 22976 |
| Min | 175 | 200 | 700 | 1881 | 22690 | 24127 | 17572 | 1003 | 18099 | 16448 | 2403 | 28248 | 20202 | 20640 | 18480 | 1950 | 21751 | 175 |
| Max | 250 | 200 | 700 | 2374 | 26729 | 29593 | 28593 | 2299 | 24426 | 29148 | 3158 | 36368 | 37663 | 33466 | 30156 | 2379 | 25597 | 37663 |

**Table 3. Summary Statistics for Time to Fever^1^, Tetracycline Start and AEs**

| **Summary Statistic** | **Time to Fever (days)** | **Time to Tetracycline Start (days)** | **Time to first Headache (days)** | **Time to first Myalgia (days)** | **Time to first Malaise (days)** | **Time to first Chest Pain (days)** | **Time to first Pyrexia (days)** | **Time to first Nausea (days)** | **Time to first Back Pain (days)** | **Time to first Vomiting (days)** | **Time to first Decreased Appetite (days)** | **Time to first Chills (days)** |
| --- | --- | --- | --- | --- | --- | --- | --- | --- | --- | --- | --- | --- |
| **200 CFU** | | | | | | | | | | | | |
| N | 6 | 6 | 5 | 5 | 3 | 2 | 0 | 2 | 0 | 2 | 4 | 1 |
| Mean | 7.2 | 7.0 | 6.0 | 6.6 | 6.7 | 7.5 | . | 7.5 | . | 7.5 | 7.0 | 5.0 |
| SD | 3.01 | 2.83 | 2.74 | 2.07 | 2.52 | 4.95 | . | 6.36 | . | 6.36 | 3.74 | . |
| Median | 6.8 | 6.5 | 6.0 | 7.0 | 7.0 | 7.5 | . | 7.5 | . | 7.5 | 6.5 | 5.0 |
| Min | 4.1 | 4.0 | 2.0 | 4.0 | 4.0 | 4.0 | . | 3.0 | . | 3.0 | 3.0 | 5.0 |
| Max | 12.4 | 12.0 | 9.0 | 9.0 | 9.0 | 11.0 | . | 12.0 | . | 12.0 | 12.0 | 5.0 |
| **700 CFU** | | | | | | | | | | | | |
| N | 4 | 4 | 4 | 4 | 4 | 2 | 0 | 1 | 1 | 1 | 3 | 0 |
| Mean | 6.8 | 7.3 | 5.0 | 6.5 | 5.3 | 7.5 | . | 4.0 | 4.0 | 5.0 | 4.3 | . |
| SD | 3.20 | 3.40 | 2.00 | 1.73 | 1.89 | 0.71 | . | . | . | . | 0.58 | . |
| Median | 5.6 | 6.5 | 4.0 | 7.0 | 4.5 | 7.5 | . | 4.0 | 4.0 | 5.0 | 4.0 | . |
| Min | 4.4 | 4.0 | 4.0 | 4.0 | 4.0 | 7.0 | . | 4.0 | 4.0 | 5.0 | 4.0 | . |
| Max | 11.4 | 12.0 | 8.0 | 8.0 | 8.0 | 8.0 | . | 4.0 | 4.0 | 5.0 | 5.0 | . |
| **2,500 CFU** | | | | | | | | | | | | |
| N | 16 | 16 | 16 | 8 | 6 | 6 | 0 | 3 | 6 | 6 | 6 | 2 |
| Mean | 4.4 | 6.6 | 3.4 | 5.0 | 4.8 | 4.8 | . | 4.0 | 4.5 | 4.7 | 4.5 | 4.5 |
| SD | 1.30 | 2.70 | 1.15 | 2.45 | 0.75 | 1.17 | . | 0.00 | 1.05 | 3.08 | 0.55 | 0.71 |
| Median | 4.1 | 5.0 | 4.0 | 4.0 | 5.0 | 4.5 | . | 4.0 | 4.5 | 4.0 | 4.5 | 4.5 |
| Min | 2.4 | 3.0 | 2.0 | 4.0 | 4.0 | 4.0 | . | 4.0 | 3.0 | 0.0 | 4.0 | 4.0 |
| Max | 8.4 | 11.0 | 6.0 | 11.0 | 6.0 | 7.0 | . | 4.0 | 6.0 | 9.0 | 5.0 | 5.0 |
| **25,000 CFU** | | | | | | | | | | | | |
| N | 85 | 85 | 84 | 42 | 37 | 30 | 43 | 27 | 20 | 16 | 10 | 18 |
| Mean | 3.1 | 3.9 | 2.7 | 3.0 | 3.6 | 3.1 | 3.1 | 3.1 | 3.2 | 3.2 | 4.4 | 2.9 |
| SD | 0.87 | 1.39 | 1.10 | 0.80 | 1.09 | 1.14 | 0.80 | 1.23 | 1.09 | 1.11 | 2.32 | 1.21 |
| Median | 3.1 | 4.0 | 3.0 | 3.0 | 3.0 | 3.0 | 3.0 | 3.0 | 3.0 | 3.0 | 3.5 | 3.0 |
| Min | 0.1 | 2.0 | 0.0 | 2.0 | 2.0 | 0.0 | 2.0 | 0.0 | 1.0 | 0.0 | 2.0 | 1.0 |
| Max | 7.1 | 9.0 | 5.0 | 5.0 | 7.0 | 7.0 | 6.0 | 6.0 | 5.0 | 5.0 | 9.0 | 7.0 |

Time to fever onset was determined for each subject using the first time point of two consecutive temperatures of 100^◦^F or greater and assumes a 9 AM challenge time. Note that this is calculated from the temperature listings as opposed to the reporting of the AE of pyrexia, which is only present in a subset of the studies.

**Table 4. Tetracycline Treatment Schedules and Efficacy**

| **Protocol #** | **Continuous Treatment** | **Subject Number** | **Exposure Date** | **Onset of Fever Date** | **Tetracycline Start Date** | **Pre-Loading Treatment on First Day** | **Tetracycline Dose (in grams) & Schedule** | **Streptomycin Start Date** | **Duration of Tetracycline Treatment (days)** | **Duration of Fever (hours)** | **Date of Loss of Fever** | **Study Day - AE >7 Days After Antibiotic Start** | **Date of Recurrence of Fever** |
| --- | --- | --- | --- | --- | --- | --- | --- | --- | --- | --- | --- | --- | --- |
| 64-06 | Yes | 64-06-0005 | 10/01/63 | 10/04/63 | 10/05/63 | Yes | 0.5 (QID) | 10/22/63 | 10 | 48 | 10/06/1963 | 19 | 10/21/1963 |
|  |  | 64-06-0014 | 10/01/63 | 10/04/63 | 10/04/63 | Yes | 0.5 (QID) | 10/27/63 | 10 | 48 | 10/06/1963 | 18 | 10/19/1963 |
|  |  | 64-06-0022 | 10/01/63 | 10/03/63 | 10/04/63 | Yes | 0.5 (QID) | 10/23/63 | 11 | 102 | 10/07/1963 | 19 | 10/22/1963 |
|  | No | 64-06-0011 | 10/01/63 | 10/04/63 | 10/04/63 | Yes | 0.5 (QID) | . | 15 | 36 | 10/05/1963 |  | . |
|  |  | 64-06-0017 | 10/01/63 | 10/03/63 | 10/04/63 | Yes | 0.5 (QID) | . | 15 | 54 | 10/05/1963 | 17 | . |
|  |  | 64-06-0018 | 10/01/63 | 10/04/63 | 10/04/63 | Yes | 0.5 (QID) | . | 15 | 30 | 10/05/1963 | 28 | . |
| 64-12 | Yes | 64-12-0001 | 02/07/64 | 02/09/64 | 02/10/64 | No | 0.5 (Q6H) | . | 21 | 72 | 02/12/1964 |  | 03/07/1964 |
|  |  | 64-12-0004 | 02/07/64 | 02/10/64 | 02/11/64 | No | 0.5 (Q6H) | . | 22 | 60 | 02/12/1964 |  | . |
|  |  | 64-12-0006 | 02/07/64 | 02/10/64 | 02/11/64 | No | 0.5 (Q6H) | . | 21 | 54 | 02/12/1964 |  | 03/07/1964 |
|  |  | 64-12-0007 | 02/07/64 | 02/10/64 | 02/10/64 | No | 0.5 (Q6H) | . | 26 | 54 | 02/12/1964 |  | 02/20/1964 |
|  |  | 64-12-0013 | 02/07/64 | 02/10/64 | 02/13/64 | Yes | 0.5 (Q6H) | . | 21 | 96 | 02/14/1964 |  | . |
|  |  | 64-12-0014 | 02/07/64 | 02/13/64 | 02/13/64 | Yes | 0.5 (Q6H) | . | 26 | 48 | 02/15/1964 |  | . |
|  |  | 64-12-0015 | 02/07/64 | 02/10/64 | 02/10/64 | Yes | 0.5 (Q6H) | . | 16 | 60 | 02/12/1964 | 23 | 02/19/1964 |
|  |  | 64-12-0017 | 02/07/64 | 02/11/64 | 02/10/64 | Yes | 0.5 (Q6H) | . | 25 | 54 | 02/13/1964 |  | 02/20/1964 |
|  |  | 64-12-0018 | 02/07/64 | 02/09/64 | 02/10/64 | Yes | 0.5 (Q6H) | . | 21 | 60 | 02/11/1964 |  | . |
|  |  | 64-12-0019 | 02/07/64 | 02/10/64 | 02/10/64 | Yes | 0.5 (Q6H) | . | 23 | 60 | 02/12/1964 |  | . |
|  |  | 64-12-0020 | 02/07/64 | 02/11/64 | 02/11/64 | Yes | 0.5 (Q6H) | . | 26 | 54 | 02/13/1964 | 34 | . |
|  |  | 64-12-0021 | 02/07/64 | 02/14/64 | 02/15/64 | Yes | 0.5 (Q6H) | . | 26 | 54 | 02/16/1964 | 18 | 03/11/1964 |
|  |  | 64-12-0022 | 02/07/64 | 02/10/64 | 02/14/64 | No | 0.5 (Q6H) | . | 21 | 102 | 02/14/1964 |  | . |
|  |  | 64-12-0023 | 02/07/64 | 02/08/64 | 02/12/64 | Yes | 0.5 (Q6H) | . | 17 | 144 | 02/14/1964 |  | 02/22/1964 |
|  |  | 64-12-0024 | 02/07/64 | 02/12/64 | 02/15/64 | Yes | 0.5 (Q6H) | . | 25 | 114 | 02/16/1964 |  | 03/05/1964 |
|  | No | 64-12-0011 | 02/07/64 | 02/10/64 | 02/10/64 | Yes | 0.5 (Q6H) | . | 47 | 72 | 02/13/1964 |  | . |
| 64-15 | Yes | 64-15-0001 | 04/09/64 | 04/13/64 | 04/13/64 | No | 0.5 (Q6H) | . | 22 | 36 | 04/14/1964 |  | . |
|  |  | 64-15-0002 | 04/09/64 | 04/12/64 | 04/12/64 | No | 0.5 (Q6H) | . | 22 | 54 | 04/14/1964 |  | . |
|  |  | 64-15-0003 | 04/09/64 | 04/12/64 | 04/12/64 | No | 0.5 (Q6H) | . | 16 | 48 | 04/14/1964 |  | . |
|  |  | 64-15-0005 | 04/09/64 | 04/12/64 | 04/12/64 | No | 0.5 (Q6H) | . | 16 | 60 | 04/14/1964 |  | . |
|  |  | 64-15-0007 | 04/09/64 | 04/13/64 | 04/14/64 | No | 0.5 (Q6H) | . | 16 | 72 | 04/16/1964 |  | . |
|  |  | 64-15-0009 | 04/09/64 | 04/13/64 | 04/16/64 | No | 0.5 (Q6H) | . | 16 | 96 | 04/17/1964 |  | . |
|  |  | 64-15-0010 | 04/09/64 | 04/12/64 | 04/13/64 | No | 0.5 (Q6H) | . | 22 | 72 | 04/15/1964 |  | . |
|  |  | 64-15-0012 | 04/09/64 | 04/12/64 | 04/12/64 | No | 0.5 (Q6H) | . | 22 | 54 | 04/14/1964 |  | . |
| 65-05 | Yes | 65-05-0001 | 08/04/64 | 08/06/64 | 08/08/64 | No | 0.5 (Q6H) | . | 11 | 72 | 08/09/1964 |  | . |
|  |  | 65-05-0002 | 08/04/64 | 08/08/64 | 08/08/64 | No | 0.5 (Q6H) | . | 11 | 54 | 08/10/1964 | 21 | . |
|  |  | 65-05-0003 | 08/04/64 | 08/08/64 | 08/08/64 | No | 0.5 (Q6H) | . | 11 | 54 | 08/10/1964 |  | . |
|  |  | 65-05-0004 | 08/04/64 | 08/07/64 | 08/08/64 | No | 0.5 (Q6H) | 08/29/64 | 11 | 78 | 08/10/1964 |  | 08/29/1964 |
|  |  | 65-05-0005 | 08/04/64 | 08/07/64 | 08/07/64 | No | 0.5 (Q6H) | . | 16 | 60 | 08/09/1964 |  | 09/01/1964 |
|  |  | 65-05-0007 | 08/04/64 | 08/07/64 | 08/08/64 | No | 0.5 (Q6H) | . | 16 | 54 | 08/09/1964 |  | . |
|  |  | 65-05-0008 | 08/04/64 | 08/07/64 | 08/08/64 | No | 0.5 (Q6H) | 08/24/64 | 11 | 48 | 08/09/1964 |  | 08/24/1964 |
|  |  | 65-05-0009 | 08/04/64 | 08/07/64 | 08/07/64 | No | 0.5 (Q6H) | . | 11 | 54 | 08/09/1964 |  | . |
|  |  | 65-05-0010 | 08/04/64 | 08/08/64 | 08/08/64 | No | 0.5 (Q6H) | . | 16 | 48 | 08/10/1964 |  | . |
|  |  | 65-05-0011 | 08/04/64 | 08/08/64 | 08/08/64 | No | 0.5 (Q6H) | . | 11 | 48 | 08/10/1964 |  | . |
|  |  | 65-05-0013 | 08/04/64 | 08/07/64 | 08/07/64 | No | 0.5 (Q6H) | . | 16 | 54 | 08/09/1964 |  | . |
|  |  | 65-05-0015 | 08/04/64 | 08/07/64 | 08/07/64 | No | 0.5 (Q6H) | . | 16 | 54 | 08/09/1964 |  | . |
|  |  | 65-05-0017 | 08/04/64 | 08/07/64 | 08/07/64 | No | 0.5 (Q6H) | . | 16 | 54 | 08/09/1964 | 15 | . |
|  |  | 65-05-0019 | 08/04/64 | 08/07/64 | 08/07/64 | No | 0.5 (Q6H) | . | 16 | 54 | 08/09/1964 | 11 | . |
|  |  | 65-05-0020 | 08/04/64 | 08/07/64 | 08/07/64 | No | 0.5 (Q6H) | . | 16 | 48 | 08/09/1964 |  | . |
|  |  | 65-05-0021 | 08/04/64 | 08/07/64 | 08/08/64 | No | 0.5 (Q6H) | . | 11 | 54 | 08/09/1964 |  | . |
| 66-01 | Yes | 66-01-0001 | 09/16/65 | 09/18/65 | 09/22/65 | No | 0.25 (Q6H) | . | 15 | 108 | 09/22/1965 |  | . |
|  |  | 66-01-0003 | 09/16/65 | 09/18/65 | 09/19/65 | No | 0.25 (Q6H) | 10/12/65 | 16 | 96 | 09/22/1965 | 24 | 10/10/1965 |
|  |  | 66-01-0004 | 09/16/65 | 09/18/65 | 09/19/65 | No | 0.25 (Q6H) | . | 17 | 78 | 09/21/1965 |  | . |
|  |  | 66-01-0006 | 09/16/65 | 09/18/65 | 09/18/65 | No | 0.25 (Q6H) | . | 16 | 54 | 09/20/1965 |  | . |
|  |  | 66-01-0007 | 09/16/65 | 09/19/65 | 09/23/65 | No | 0.25 (Q6H) | . | 16 | 150 | 09/25/1965 |  | . |
|  |  | 66-01-0009 | 09/16/65 | 09/18/65 | 09/19/65 | No | 0.25 (Q6H) | . | 16 | 78 | 09/21/1965 |  | . |
|  |  | 66-01-0011 | 09/16/65 | 09/18/65 | 09/18/65 | No | 0.25 (Q6H) | 10/15/65 | 16 | 72 | 09/21/1965 |  | 10/14/1965 |
|  |  | 66-01-0014 | 09/16/65 | 09/18/65 | 09/19/65 | No | 0.25 (Q6H) | . | 16 | 90 | 09/21/1965 |  | . |
| 66-13 | Yes | 66-13-0001 | 01/25/66 | 01/29/66 | 01/29/66 | No | 1 (BID) | . | 15 | 54 | 01/31/1966 |  | . |
|  |  | 66-13-0002 | 01/25/66 | 01/28/66 | 01/28/66 | No | 1 (BID) | . | 15 | 54 | 01/30/1966 |  | . |
|  |  | 66-13-0003 | 01/25/66 | 01/28/66 | 01/29/66 | Yes | 1 (QID) | . | 16 | 54 | 01/30/1966 |  | . |
|  |  | 66-13-0004 | 01/25/66 | 01/28/66 | 01/29/66 | Yes | 0.5 (QID) | . | 17 | 54 | 01/30/1966 |  | . |
|  |  | 66-13-0006 | 01/25/66 | 01/27/66 | 01/28/66 | Yes | 0.5 (QID) | . | 17 | 90 | 01/30/1966 |  | . |
|  |  | 66-13-0007 | 01/25/66 | 01/28/66 | 01/28/66 | No | 1 (BID) | . | 15 | 48 | 01/30/1966 |  | . |
|  |  | 66-13-0009 | 01/25/66 | 01/28/66 | 01/29/66 | Yes | 0.5 (QID) | . | 16 | 78 | 01/31/1966 |  | . |
|  |  | 66-13-0010 | 01/25/66 | 01/28/66 | 01/29/66 | Yes | 0.5 (QID) | . | 15 | 54 | 01/30/1966 |  | . |
|  |  | 66-13-0012 | 01/25/66 | 01/29/66 | 01/29/66 | Yes | 0.5 (QID) | 02/01/66 | 4 | 42 | 01/30/1966 | 16 | 02/04/1966 |
|  |  | 66-13-0013 | 01/25/66 | 01/28/66 | 01/29/66 | No | 1 (BID) | . | 14 | 78 | 01/31/1966 | 12 | . |
|  |  | 66-13-0015 | 01/25/66 | 01/28/66 | 01/29/66 | Yes | 0.5 (QID) | . | 16 | 60 | 01/30/1966 |  | . |
|  |  | 66-13-0016 | 01/25/66 | 01/28/66 | 01/29/66 | No | 1 (BID) | . | 14 | 54 | 01/30/1966 |  | . |
|  |  | 66-13-0018 | 01/25/66 | 01/28/66 | 01/29/66 | No | 1 (BID) | . | 15 | 72 | 01/31/1966 |  | . |
|  | No | 66-13-0008 | 01/25/66 | 01/25/66 | 01/30/66 | Yes | 0.5 (QID) | . | 15 | 132 | 01/30/1966 |  | . |
